# Supplementary material for: Enhanced Ca2+-channeling complex formation at the ER-mitochondria interface underlies the pathogenesis of alcohol-associated liver disease
Source: Nat Commun. 2023 Mar 27;14:1703. doi: 10.1038/s41467-023-37214-4 (PMC10042999; doi:10.1038/s41467-023-37214-4)
Supplement: Supplementary file 1 — Supplementary Information [file 41467_2023_37214_MOESM1_ESM.pdf]

Supplementary Information

Enhanced Ca^{2+}-channeling complex formation at the ER-mitochondria interface underlies the pathogenesis of alcohol-associated liver disease

Themis Thoudam[1], Dipanjan Chanda[1,2], Jung Yi Lee[2], Min-Kyo Jung[3], Ibotombi Singh Sinam[4], Byung-Gyu Kim[5], Bo-Yoon Park[1], Woong Hee Kwon[2], Hyo-Jeong Kim[6], Myeongjin Kim[1,7], Chae Won Lim[4,7], Hoyul Lee[1], Yang Hoon Huh[6], Caroline A. Miller[8], Romil Saxena[9,10], Nicholas J Skill[11], Nazmul Huda[12], Praveen Kusumanchi[12], Jing Ma[12], Zhihong Yang[12], Min-Ji Kim[13], Ji Young Mun[3], Robert A Harris[14], Jae-Han Jeon[15], Suthat Liangpunsakul[12,14,16]*, In-Kyu Lee[1,17]*

[1]Research Institute of Aging and Metabolism, Kyungpook National University, Daegu, Republic of Korea; [2]Leading-Edge Research Center for Drug Discovery and Development for Diabetes and Metabolic Disease, Kyungpook National University Hospital, Daegu, Republic of Korea; [3]Neural Circuit Research Group, Korea Brain Research Institute, Daegu, Republic of Korea; [4]Bio-Medical Research Institute, Kyungpook National University Hospital, Daegu, Daegu, Republic of Korea; [5]Center for Genomic Integrity, Institute for Basic Science (IBS), Ulsan, Republic of Korea; [6]Electron Microscopy Research Center, Korea Basic Science Institute, Ochang, Chungbuk, Republic of Korea; [7]Department of Medicine, Daegu Catholic University, Daegu, Republic of Korea; [8]Electron Microscopy Core, Indiana University School of Medicine, Indianapolis, Indiana, USA; [9]Department of Pathology and Laboratory Medicine, Indiana University School of Medicine, Indianapolis, Indiana, USA; [10]Department of Pathology and Laboratory Medicine, Emory University School of Medicine, Atlanta, Georgia, USA; [11]Department of Surgery, Louisiana State University Health Science Center, New Orleans, Louisiana, USA; [12]Division of Gastroenterology and Hepatology, Department of Medicine, Indiana University School of Medicine, Indianapolis, Indiana, USA; [13]Department of Internal Medicine, Kyungpook National University Chilgok Hospital, Daegu, Republic of Korea; [14]Department of Biochemistry and Molecular Biology, Indiana University School of Medicine, Indianapolis, Indiana, USA; [15]Department of Internal Medicine, School of Medicine, Kyungpook National University, Kyungpook National University Chilgok Hospital, Daegu, Republic of Korea; [16]Richard L. Roudebush VA Medical Center, Indianapolis, Indiana, USA; [17]Department of Internal Medicine, School of Medicine, Kyungpook National University, Kyungpook National University Hospital, Daegu, Republic of Korea.

*To whom correspondence should be addressed:
leei@knu.ac.kr and sliangpu@iu.edu

Contents:

Supplementary Figures 1-10
Supplementary Tables 1-4

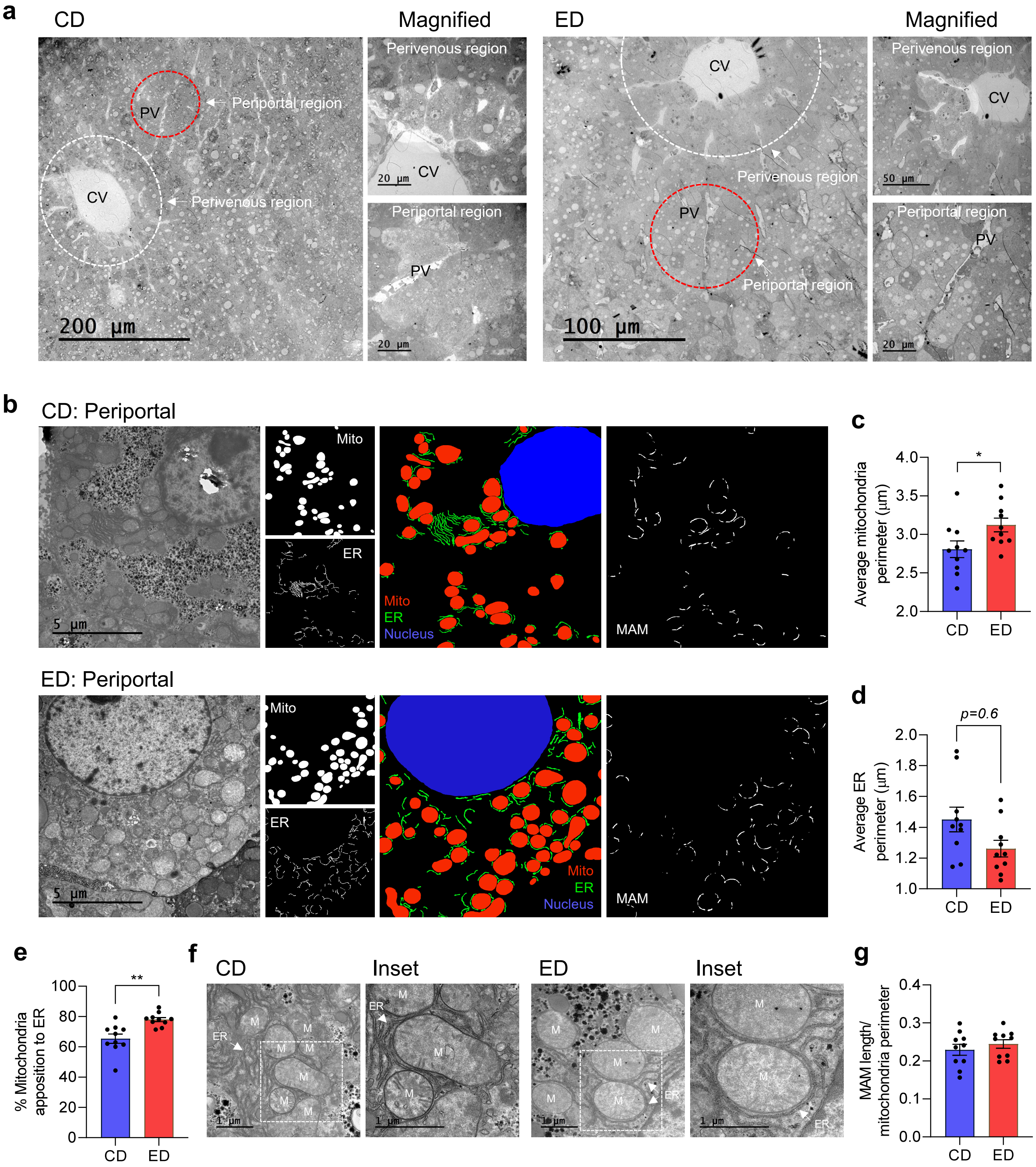
a
CD
Magnified
ED
Magnified
PV
Periportal region
CV
Perivenous region
200 µm
Perivenous region
20 µm
Periportal region
PV
20 µm
CV
Perivenous region
PV
Periportal region
100 µm
50 µm
20 µm
b
CD: Periportal
5 µm
Mito
ER
Mito
ER
Nucleus
MAM
ED: Periportal
5 µm
Mito
ER
Mito
ER
Nucleus
MAM
c
*
Average mitochondria perimeter (µm)
4.0
3.5
3.0
2.5
2.0
CD
ED
d
p=0.6
Average ER perimeter (µm)
2.0
1.8
1.6
1.4
1.2
1.0
CD
ED
e
**
% Mitochondria apposition to ER
100
80
60
40
20
0
CD
ED
f
CD
Inset
ED
Inset
ER
M
1 µm
g
MAM length/ mitochondria perimeter
0.4
0.3
0.2
0.1
0.0
CD
ED

Supplementary Fig 1. Alcohol increases the ER-mitochondrial contact sites, but not the length in the hepatic periportal region.

a Demonstration of perivenous (white doted circles) and periportal (red dotted circles) region mapping under EM for imaging and analysis of hepatocyte populations within 50μm from the central vein (CV) or portal vein (PV) border. **b** Mitochondria and ER morphology in the periportal region of CD and ED-fed mice liver sections were visualized using TEM (Scale bars, 5μm). The ER and mitochondria in the TEM images were reconstructed graphically to visualize MAM formation. Mito: Mitochondria (red), ER: Endoplasmic Reticulum (green), Nucleus (blue). **c-g** Quantification of average mitochondria perimeter (**c**), ER perimeter (**d**), percentage of mitochondria apposition to ER (**e**), magnified TEM images (Scale bars, 1μm) (M: Mitochondria, ER: Endoplasmic Reticulum) (**f**), and MAM length to mitochondrial perimeter ratio (**g**) (n=10 microscopic fields each; number of mitochondria/ER analyzed, CD: 160/460 and ED: 230/680 from 3 mice/group). All values are represented as mean ± SEM. *$p<0.05$; **$p<0.01$ (Two-tailed unpaired t-test).

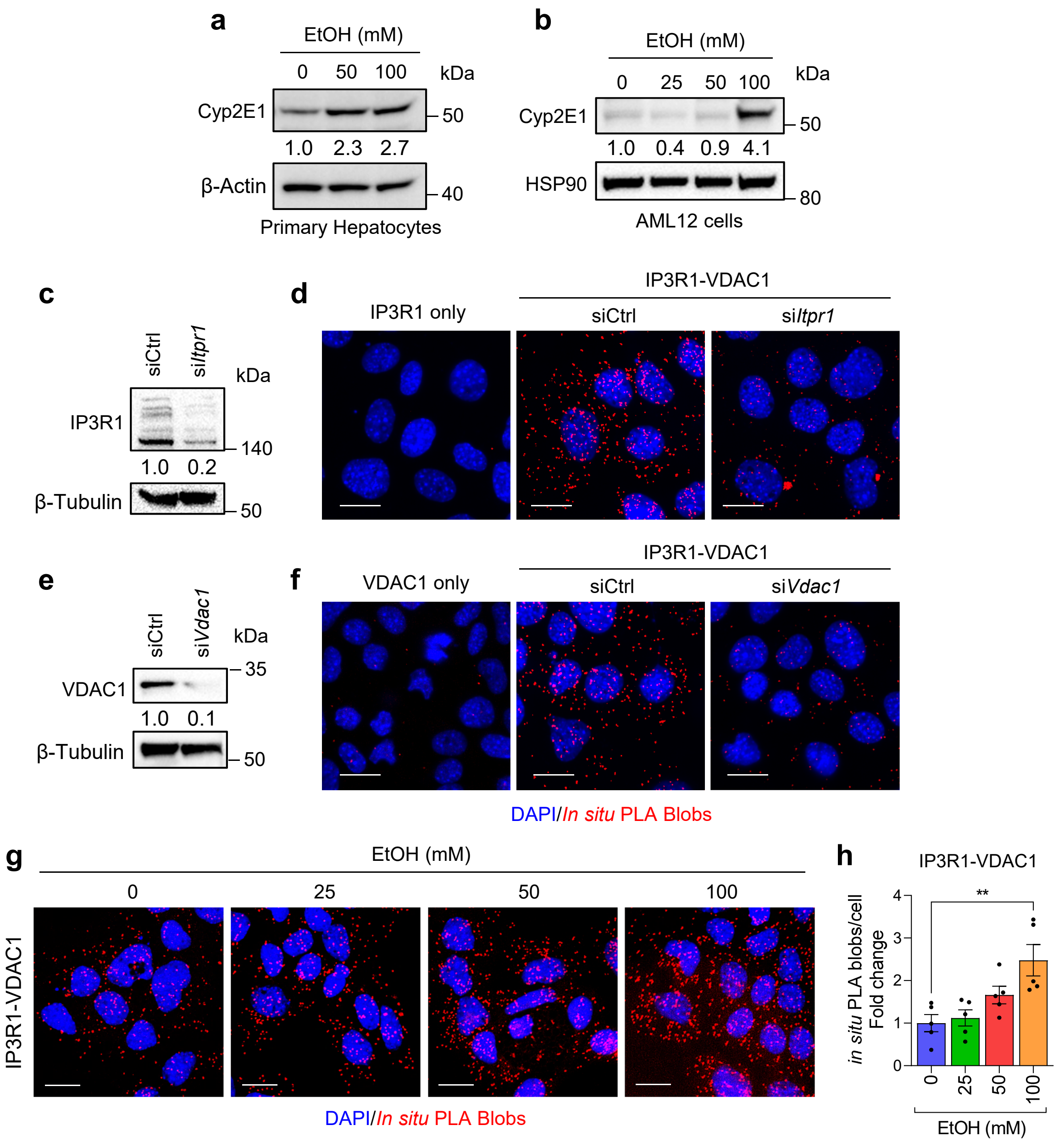

Supplementary Fig 2. EtOH induces MCC complex formation.
a, b Evaluation of Cyp2E1 expression by immunoblotting in primary hepatocytes (**a**) and AML12 cells (**b**) treated with the indicated doses of EtOH for 24h. **c, d** Evaluation of IP3R1 expression (**c**) and IP3R1-VDAC1 interaction (Scale bars, 20μm) (**d**) in AML12 cells transfected with siCtrl or si*Itpr1* for 48h by immunoblotting and *in situ* PLA, respectively. **e, f** Evaluation of VDAC1 expression (**e**), and IP3R1-VDAC1 interactions (Scale bars, 20μm) (**f**) in AML12 cells transfected with siCtrl or si*Vdac1* for 48h by immunoblotting and *in situ* PLA, respectively. *in situ* PLA with a single antibody served as a negative control. **g** IP3R1-VDAC1 interaction analysis by *in situ* PLA in AML12 cells treated with the indicated doses of EtOH for 24h (Scale bars, 20μm). **h** Quantification of *in situ* PLA shown in (**g**) (n=5 microscopic fields with >200 cells from 3 independent experiments). All quantifications are represented as mean ± SEM, ***p*<0.01 (Ordinary one-way ANOVA, Dunnett's multiple comparisons test). Quantifications relative to control are provided below each blot.

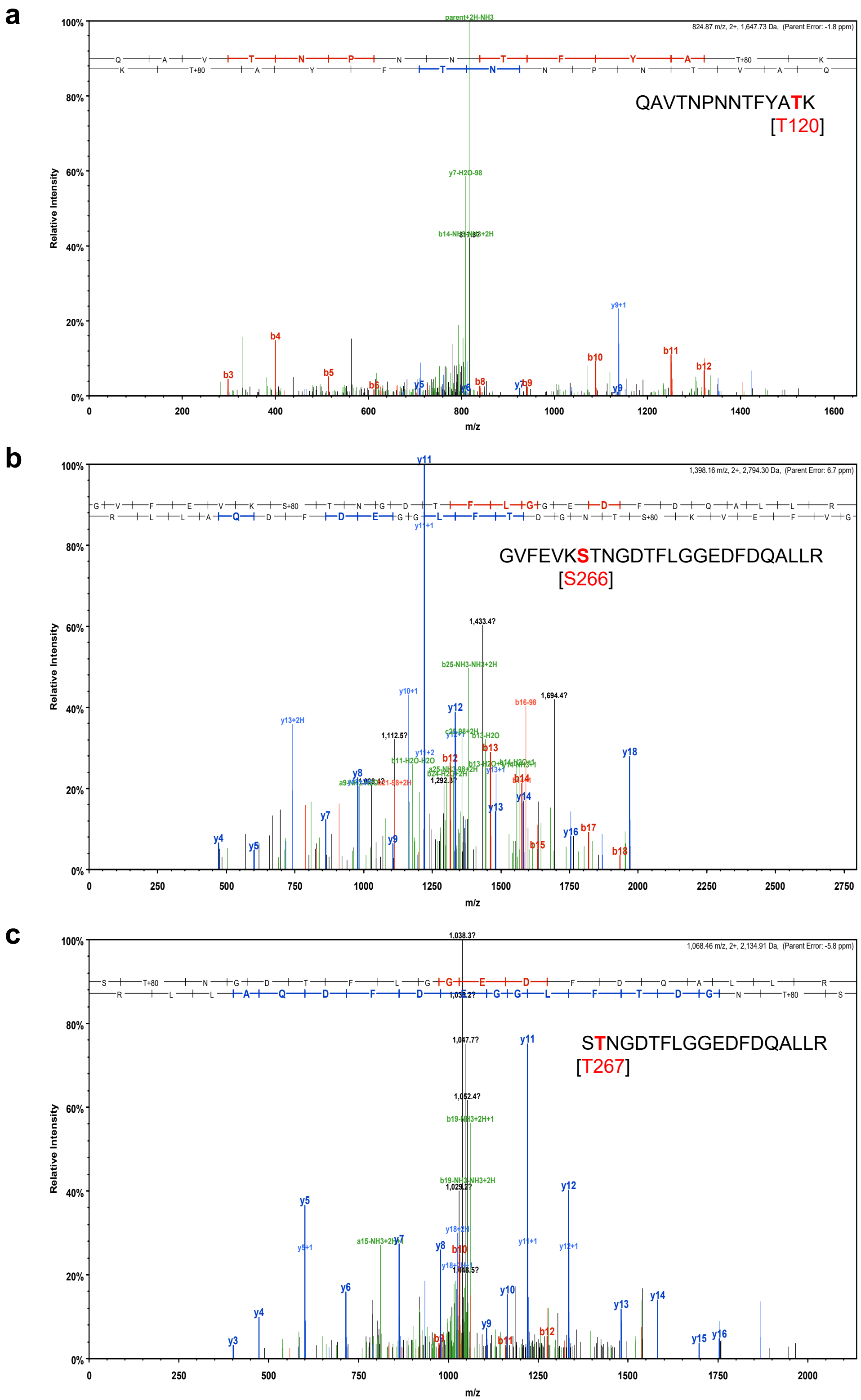

Supplementary Fig 3. PDK4 phosphorylates GRP75 at multiple sites.
a-c Detection of GRP75 phosphopeptides by LC-MS/MS analysis. Thr240 [T120] (**a**), Ser266 [S266] (**b**), and Thr267 [T267] (**c**) of GRP75 are phosphorylated by PDK4.

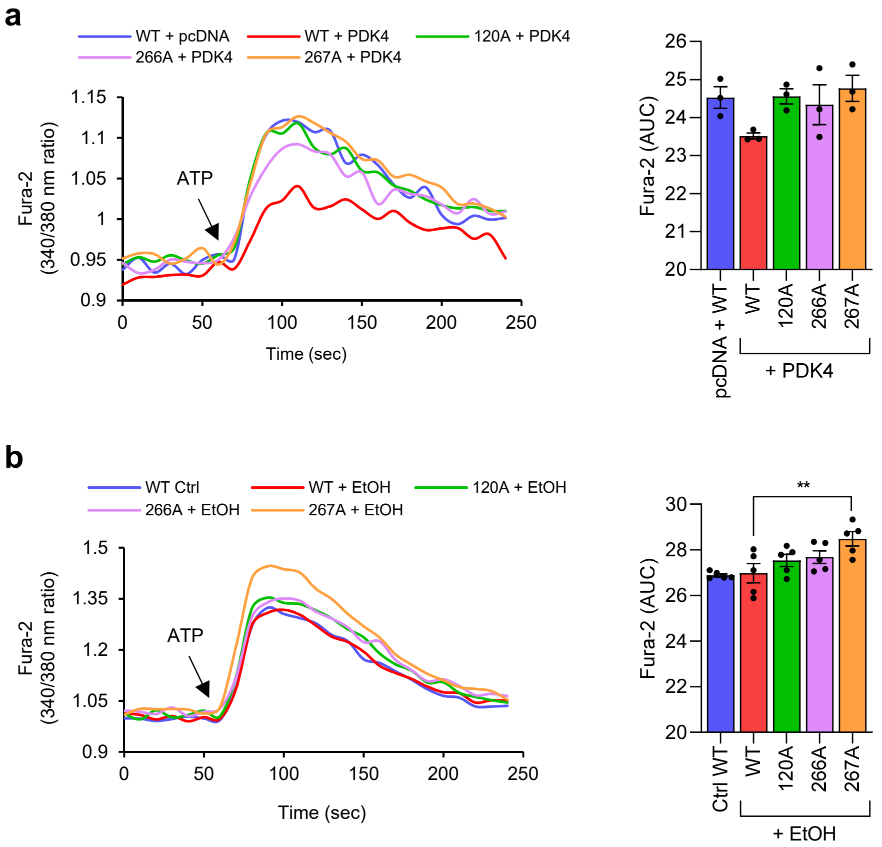

Supplementary Fig 4. GRP75 phosphorylation does not affect IP3R-mediated Ca^{2+} release.
a Measurement of cytosolic Ca^{2+} flux in AML12 cells co-overexpressing PDK4 and WT GRP75 or phospho-mutant GRP75 using Fura-2 ratiometric dye. 100uM ATP was injected 60 seconds later to stimulate the IP3R-mediated Ca^{2+} release and quantification of area under the curve (AUC) (right panel) (n=3 biological replicates from 2 independent experiment). **b** Measurement of cytosolic Ca^{2+} flux using Fura-2 ratiometric dye in AML12 cells overexpressing WT or phospho-mutant GRP75 and treated with 100mM EtOH for 24h. 100uM ATP was injected 60 seconds later to stimulate the IP3R-mediated Ca^{2+} release and quantification of AUC (right panel) (n=5 biological replicates from 2 independent experiment). All quantifications are represented as mean ± SEM, (Ordinary one-way ANOVA, Dunnett's multiple comparisons test).

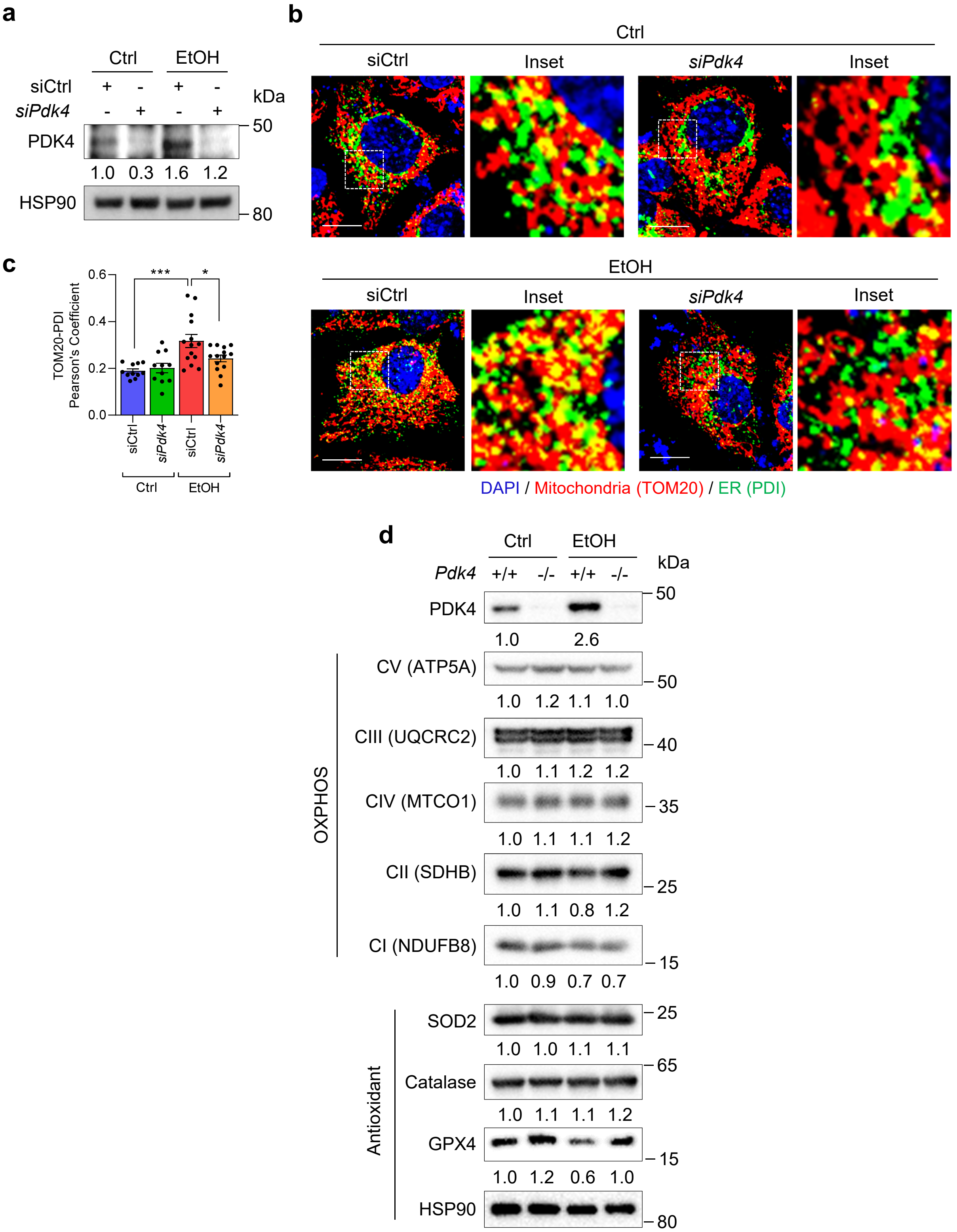
a
Ctrl
EtOH
siCtrl
siPdk4
+ - + -
- + - +
kDa
PDK4
50
1.0 0.3 1.6 1.2
HSP90
80
b
Ctrl
siCtrl
Inset
siPdk4
Inset
EtOH
siCtrl
Inset
siPdk4
Inset
DAPI / Mitochondria (TOM20) / ER (PDI)
c
TOM20-PDI
Pearson's Coefficient
0.6
0.4
0.2
0.0
***
*
siCtrl
siPdk4
siCtrl
siPdk4
Ctrl
EtOH
d
Ctrl
EtOH
Pdk4 +/+ -/- +/+ -/-
kDa
PDK4
50
1.0 2.6
OXPHOS
CV (ATP5A)
50
1.0 1.2 1.1 1.0
CIII (UQCRC2)
40
1.0 1.1 1.2 1.2
CIV (MTCO1)
35
1.0 1.1 1.1 1.2
CII (SDHB)
25
1.0 1.1 0.8 1.2
CI (NDUFB8)
15
1.0 0.9 0.7 0.7
Antioxidant
SOD2
25
1.0 1.0 1.1 1.1
Catalase
65
1.0 1.1 1.1 1.2
GPX4
15
1.0 1.2 0.6 1.0
HSP90
80

Supplementary Fig 5. PDK4 knockdown suppresses MAM formation in AML12 cells.
a Immunoblot analysis of PDK4 expression in AML12 cells transfected with siCtrl or si*Pdk4* and treated with or without 100mM EtOH for 24h. **b** Immunofluorescence imaging of ER (green) and mitochondria (red) using PDI and TOM20 antibodies, respectively, in AML12 cells transfected with siCtrl or si*Pdk4* and treated with 100mM EtOH for 24h (Scale bars, 10μm). **c** Quantification of TOM20-PDI colocalization shown in (**b**) (siCtrl/si*Pdk4* Ctrl, n=11; siCtrl/si*Pdk4* EtOH, n=14 microscopic fields with >300 cells from 3 independent experiments). **d** Expression of the indicated antioxidant and mitochondrial OXPHOS complex proteins were analyzed by immunoblot in primary hepatocytes treated with or without EtOH for 24h. All quantifications are represented as mean ± SEM, **p*<0.05; ****p*<0.001 (Two-way ANOVA, Tukey's multiple comparisons test). Quantifications relative to control are provided below each blot.

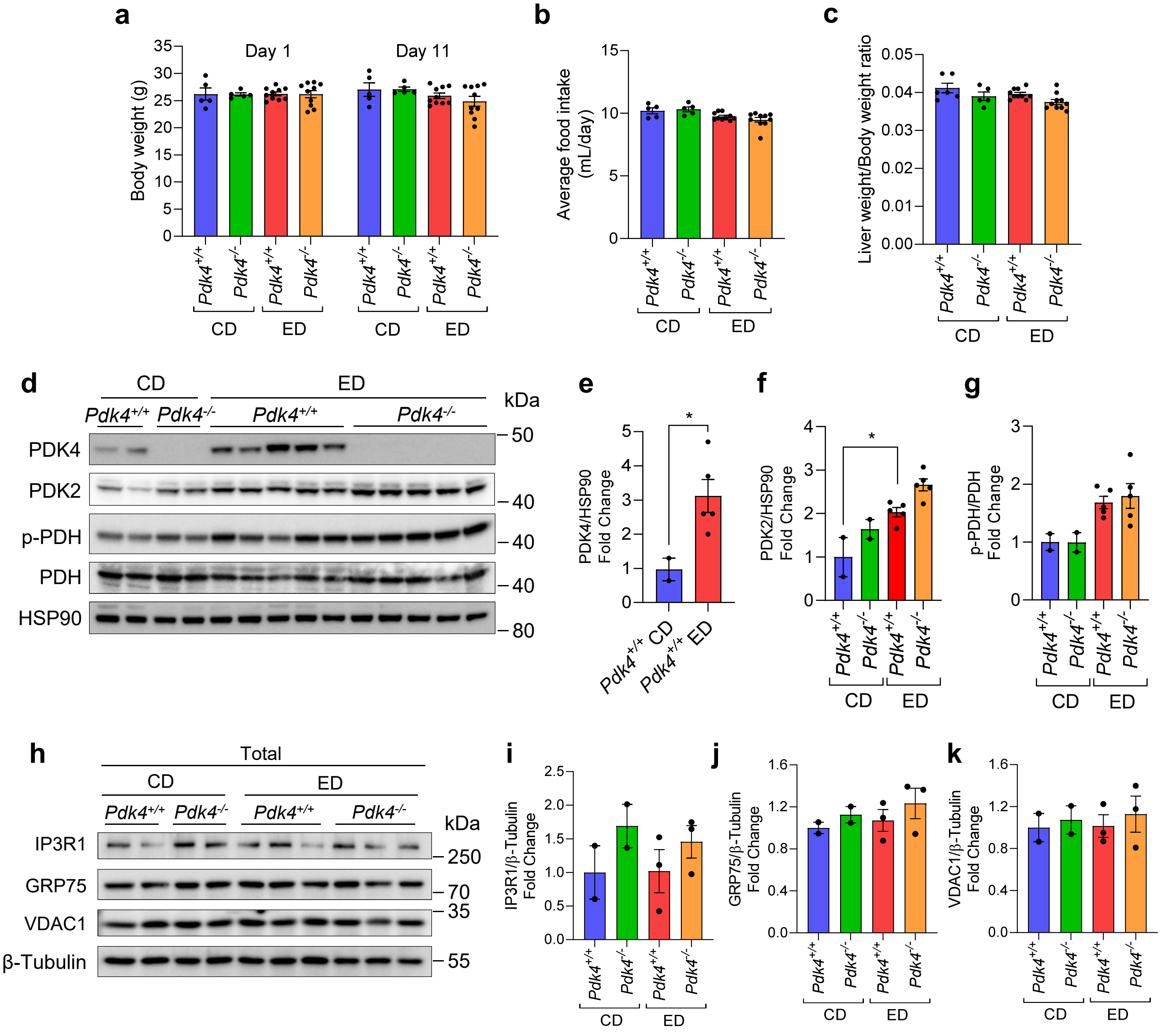

Supplementary Fig 6. PDK4 deficiency failed to suppress alcohol-induced PDH phosphorylation.
a-c Body weight in gram (g) (**a**), average food intake per mouse in mL during the CD/ED-feeding period (**b**), and liver weight to body weight ratio (**c**) (CD *Pdk4*$^{+/+}$/*Pdk4*$^{-/-}$, n=5; ED *Pdk4*$^{+/+}$/*Pdk4*$^{-/-}$, n=10 mice/group). **d-g** Immunoblot analysis of the indicated proteins (**d**), and quantifications (CD *Pdk4*$^{+/+}$/*Pdk4*$^{-/-}$, n=2; ED *Pdk4*$^{+/+}$/*Pdk4*$^{-/-}$, n=5 mice/group) (**e-g**). **h-k** Total protein expression of MAM Ca^{2+} channeling complex were evaluated by immunoblotting (**h**), and quantifications (**i-k**) (CD *Pdk4*$^{+/+}$/*Pdk4*$^{-/-}$, n=2; ED *Pdk4*$^{+/+}$/*Pdk4*$^{-/-}$, n=3 mice/group). All quantifications are represented as mean ± SEM, *$p<0.05$; **$p<0.01$ (Two-way ANOVA, Tukey's multiple comparisons test or two-tailed unpaired t-test).

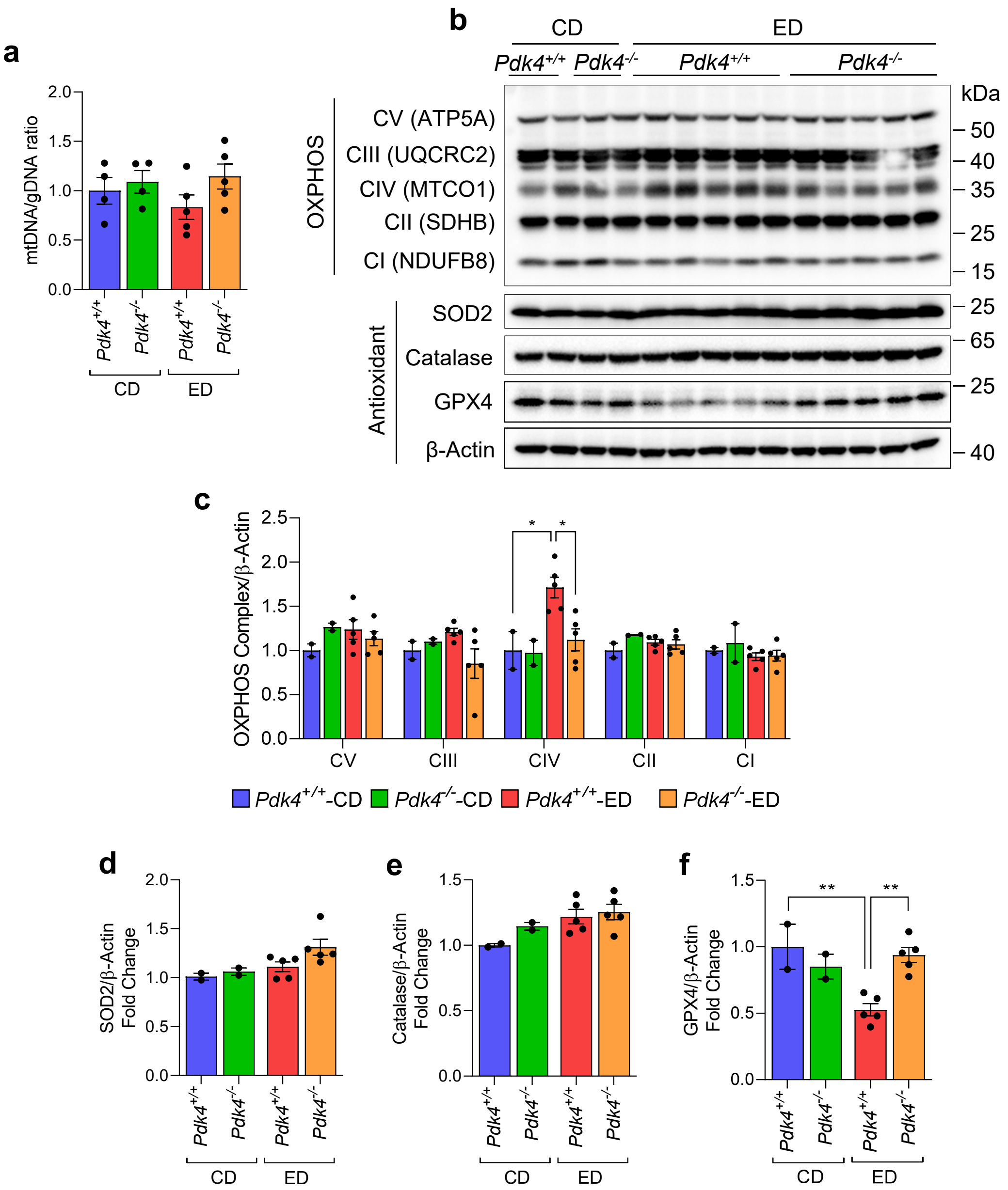

Supplementary Fig 7. Effect of PDK4 deficiency on antioxidant and OXPHOS protein expression.
a Quantification of mtDNA (mitochondrial ND1) content, normalized by gDNA (nuclear Pecam1) in CD or ED-fed *Pdk4*$^{+/+}$ or *Pdk4*$^{-/-}$ mice liver (CD *Pdk4*$^{+/+}$/*Pdk4*$^{-/-}$, n=4; ED *Pdk4*$^{+/+}$/*Pdk4*$^{-/-}$, n=5 mice). **b-f** Immunoblot analysis of the indicated proteins (**b**), and quantifications (**c-f**) (CD *Pdk4*$^{+/+}$/*Pdk4*$^{-/-}$, n=2; ED *Pdk4*$^{+/+}$/*Pdk4*$^{-/-}$, n=5 mice). All quantifications are represented as mean ± SEM, **p*<0.05; **p<0.01 (Two-way ANOVA, Tukey's multiple comparisons test).

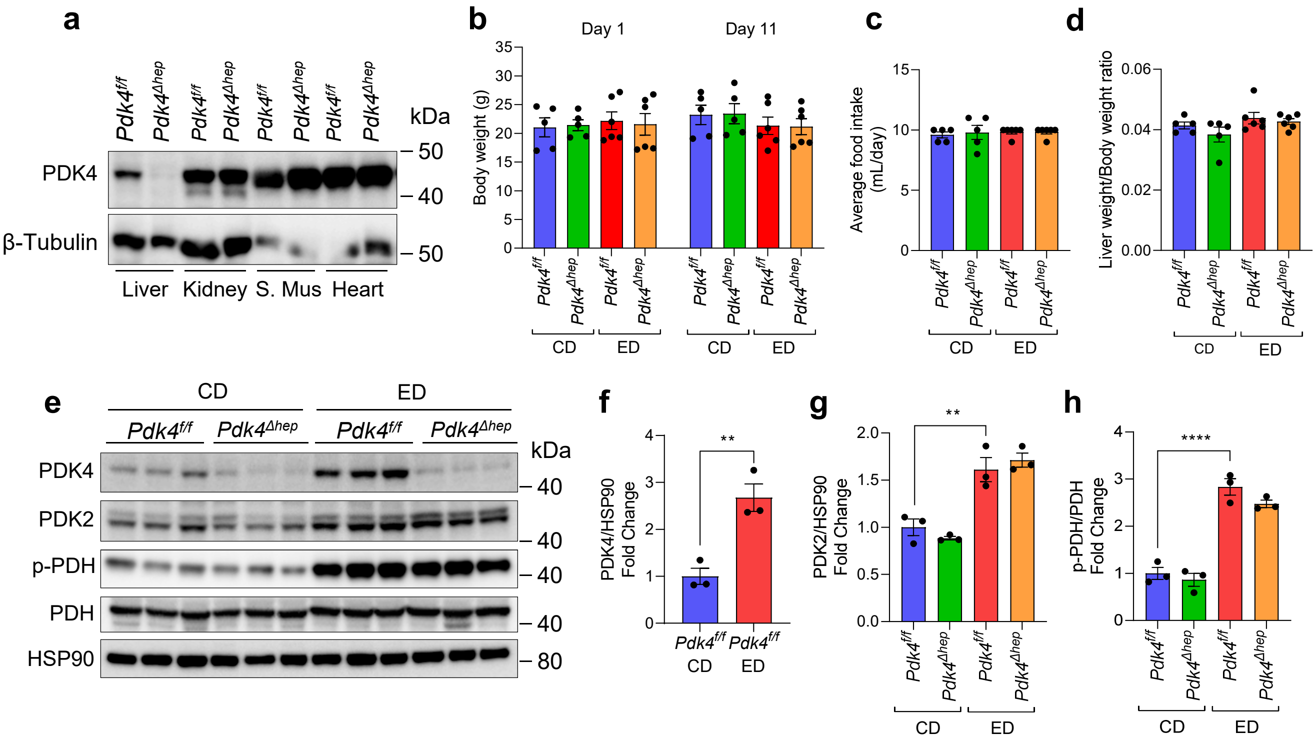

Supplementary Fig 8. Hepatocyte-specific PDK4 ablation failed to suppress alcohol-induced PDH phosphorylation.
a Evaluation of PDK4 expression in liver, kidney, gastrocnemius skeletal muscle (Sk. Mus), heart tissues from *Pdk4*$^{f/f}$ and *Pdk4*$^{\Delta hep}$ by immunoblotting. **b-d** Body weight in gram (g) (**b**), average food intake per mouse in mL during the CD/ED-feeding period (**c**), and liver weight to body weight ratio (**d**) (CD *Pdk4*$^{f/f}$/*Pdk4*$^{\Delta hep}$, n=5; ED *Pdk4*$^{f/f}$/*Pdk4*$^{\Delta hep}$, n=6 mice). **e-h** Immunoblot analysis of the indicated proteins (**e**) and quantifications (**f-h**) (CD *Pdk4*$^{f/f}$/*Pdk4*$^{\Delta hep}$, n=3; ED *Pdk4*$^{f/f}$/*Pdk4*$^{\Delta hep}$, n=3 mice). All quantifications are represented as mean ± SEM, ***p*<0.01; *****p*<0.0001 (Two-way ANOVA, Tukey's multiple comparisons test or two-tailed unpaired t-test).

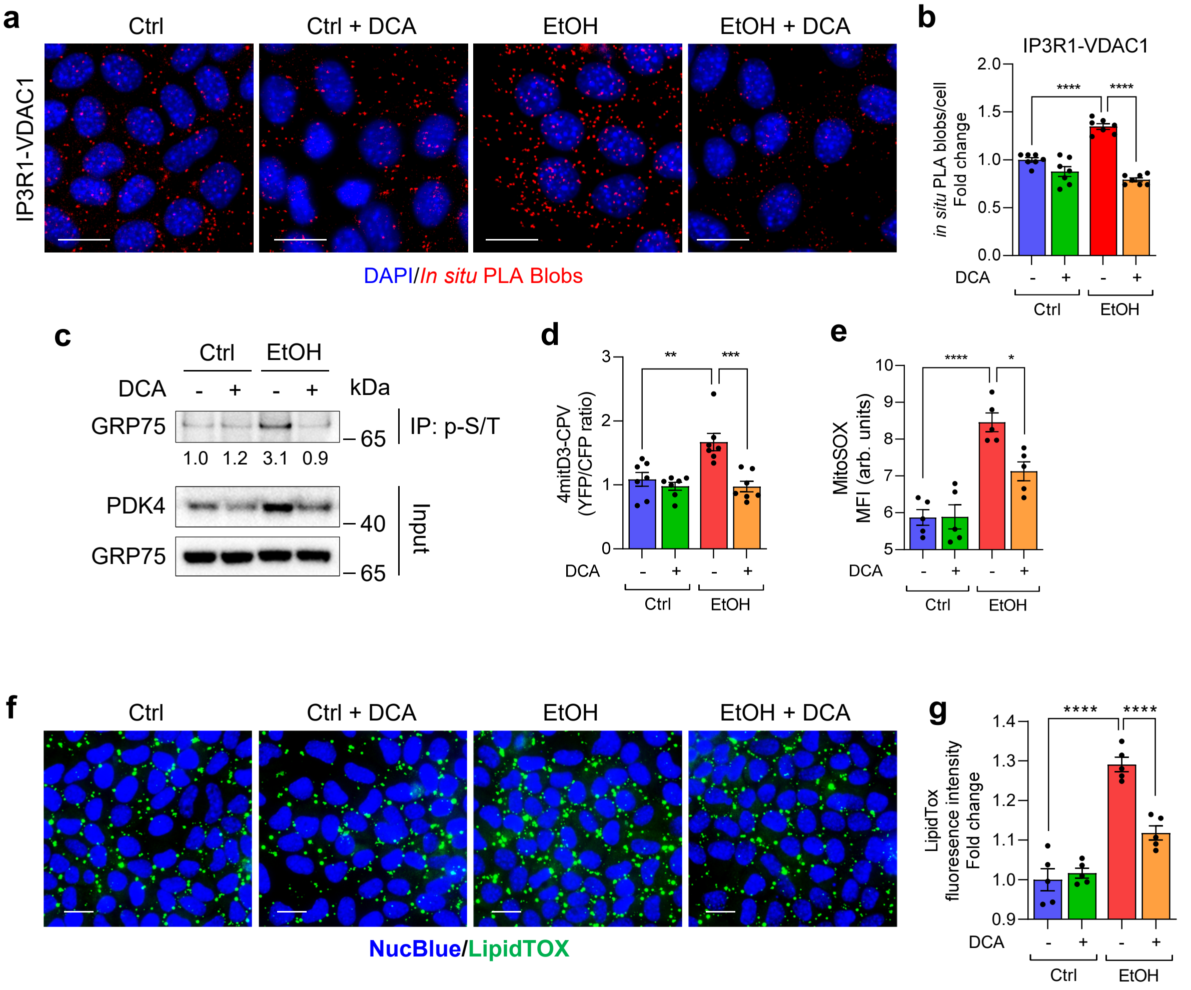

Supplementary Fig 9. Dichloroacetic acid suppresses EtOH-induced MCC complex formation and lipid accumulation.

a Analysis of IP3R1-VDAC1 interaction in AML12 cells treated with 100mM EtOH for 24h in absence or presence of 2mM DCA by *in situ* PLA (Scale bars, 20μm). **b** Quantification of *in situ* PLA shown in (**a**), (n=7 microscopic field with >500 cells from 3 independent experiments). **c** Evaluation of GRP75 phosphorylation in AML12 cells treated with EtOH for 24h in absence or presence of DCA by IP with anti-p-S/T and immunoblotting. Quantification relative to control is provided below the blot. **d** Mitochondrial Ca^{2+} level was measured after treatment of EtOH for 24h in absence or presence of DCA in AML12 cells stably expressing 4MitD3-CPV (n=7 biological replicates from 3 independent experiments). **e** Measurement of mitochondria ROS (mean fluorescence intensity; MFI) using MitoSOX dye in AML12 cells treated with EtOH for 24h in absence or presence of DCA (n=5 biological replicates from 3 independent experiments). **f** Lipid accumulation was analyzed using LipidTox dye in AML12 cells treated with EtOH for 24h in absence or presence of DCA (Scale bars, 20μm). **g** Quantification of fluorescence intensity of (**f**) (n=5 microscopic fields with >500 cells from 3 independent experiments). All quantifications are represented as mean ± SEM, **p*<0.05; ***p*<0.01; ****p*<0.001; *****p*<0.0001 (Two-way ANOVA, Tukey's multiple comparisons test).

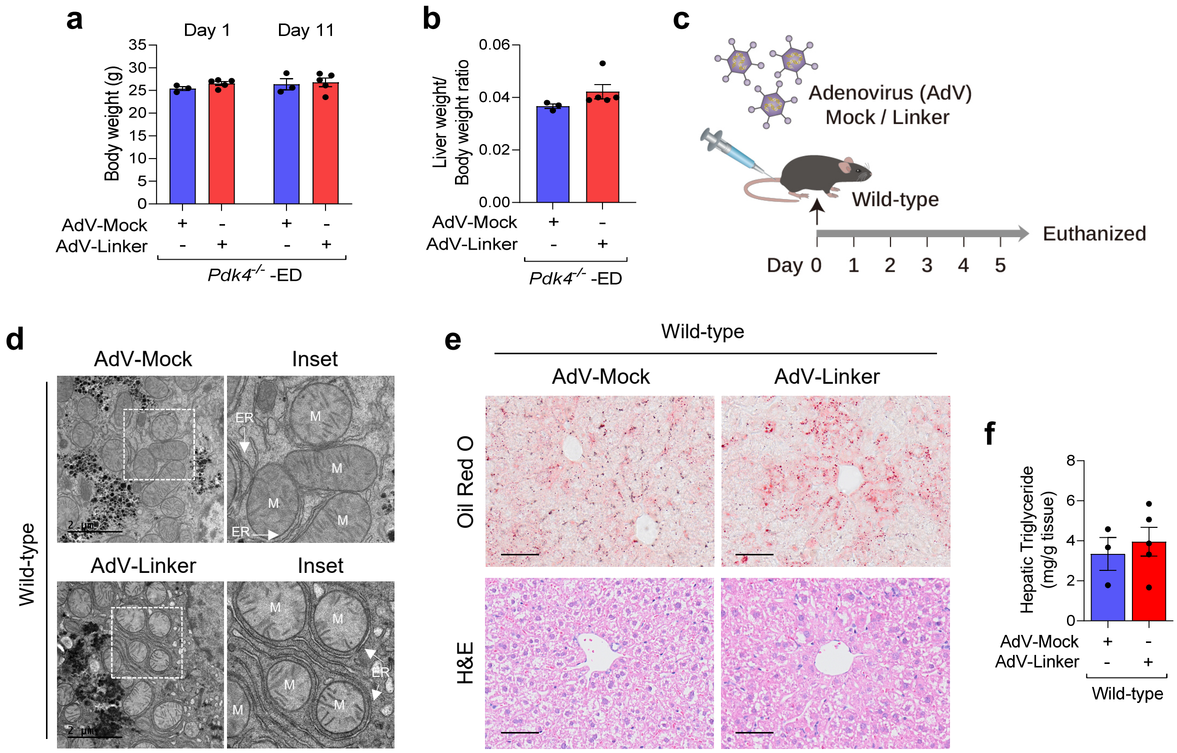

Supplementary Fig 10. Linker-mediated induction of MAM formation does not promote hepatic steatosis in absence of alcohol.

a, b Body weight in gram (g) (**a**), and liver weight to body weight ratio (**b**) of AdV-Mock/AdV-Linker injected *Pdk4*$^{-/-}$ mice-fed with ED (AdV-Mock, n=3; AdV-Linker, n=5 mice). **c** Graphical demonstration of AdV-Mock/AdV-Linker delivery by intravenous (IV) injection in wild-type mice to induce MAM formation. Mice were euthanized 5 days after the AdV injection. **d** Representative TEM images of AdV-Mock/AdV-Linker injected mice liver sections (n=2 mice/group) (Scale bars, 2µm). **e, f** Lipid accumulation and histologic changes were examined by Oil-Red-O (upper panel) and H&E (lower panel) staining, respectively (**e**) (Scale bars, 50µm), and triglyceride content (**f**) in AdV-Mock/AdV-Linker injected mice liver (AdV-Mock, n=3; AdV-Linker, n=5 mice). All quantifications are represented as mean ± SEM (Two-tailed unpaired t-test).

Supplementary Table 1. Expression of MAM-associated genes (GEO155830) in **Fig. 2a, 2b**

**MAM-associated genes**	**Log$_2$ FC**	**-Log$_{10}$ (*p-value*)**	**MAM-associated genes**	**Log$_2$ FC**	**-Log$_{10}$ (*p-value*)**
Pdk4	1.4864	4.30103	*Park7*	0.159126	0.302901
Osbpl8	-1.0129	4.30103	*Rhot1*	0.163956	0.294992
Cdip1	1.11063	3.60206	*Tchp*	-0.19969	0.261338
Tgm2	1.14271	3.522879	*Rmdn3*	0.133282	0.244201
Atf6	0.774182	2.5157	*Dnm1l*	0.132032	0.237359
Nox4	-0.81527	2.361511	*Mief1*	0.131394	0.232139
Pml	-0.7841	2.225483	*Pacs2*	-0.14539	0.227165
Psen2	-0.69134	2.177178	*Tspo*	0.121059	0.218029
Wfs1	0.727772	2.151811	*Cav1*	-0.19009	0.203044
Stat3	0.928834	2.139662	*Vapb*	-0.11597	0.199214
Ero1l	0.630824	2.094204	*Ahcyl1*	0.119629	0.190743
Bcl2l1	0.750004	2.083546	*Sepn1*	-0.14372	0.168834
Inf2	0.715995	2.024568	*Akt3*	0.196963	0.164183
Kras	0.595915	1.931814	*Trp53*	0.11823	0.160333
Fus	0.568057	1.768276	*Star*	-0.13506	0.155554
Atp2a3	-0.90584	1.693575	*Mfn2*	-0.09677	0.145967
Bok	-0.77082	1.620694	*Akt2*	-0.08804	0.136885
Nras	0.513363	1.516413	*Rtn4*	-0.08439	0.122197
Ern1	-0.51784	1.497573	*Atg14*	-0.06373	0.10251
Sec61a1	0.607241	1.479516	*Akt1*	0.063416	0.10048
Sigmar1	-0.64624	1.367543	*Atad3a*	0.055529	0.087698
Itpr1	0.572878	1.354087	*Bap1*	0.048567	0.075411
Ppp2ca	0.475323	1.309804	*Glb1*	-0.0688	0.074224
Pdzd8	0.440017	1.246034	*Canx*	-0.06162	0.07186
App	0.456088	1.125228	*Mtor*	0.046629	0.069306
Atp2a2	0.543539	1.058737	*Mfn1*	0.040135	0.062607
Mcu	-0.39734	0.880085	*Becn1*	-0.03573	0.054655
Tmx1	0.351459	0.859021	*Mief2*	0.024969	0.036189
Eif2ak3	0.379147	0.794931	*Mcl1*	0.021504	0.029653
Gsk3b	0.344161	0.793984	*Itpr3*	0.023764	0.027705
Hk2	-0.45112	0.744486	*Fis1*	-0.01333	0.01902
Hras	-0.30015	0.66374	*Pten*	-0.0112	0.01334
Ppid	0.29239	0.657183	*Tardbp*	0.012277	0.011129
Hspa5	0.520388	0.635355	*Vps13c*	-0.00821	0.010973
Hspa9	0.36121	0.603975	*Bcap31*	0.007366	0.008752
Psen1	0.282289	0.585695	*Ryr2*	1.08764	0
Rictor	0.275903	0.529002	*Fate1*	0	0
Vps13a	0.252095	0.516698	*Reep1*	-0.1142	0
Bax	-0.27777	0.475864	*Prrx1*	-0.46237	0
Bak1	0.336301	0.466673	*Tespa1*	-0.48231	0
Itpr2	-0.23437	0.429574	*Ncs1*	-0.53217	0
Snca	-0.39724	0.412514	*Ryr1*	-0.56758	0
Brca1	-0.33805	0.407435	*Atp2a1*	-0.58834	0
Mff	0.263037	0.401812	*Ryr3*	-0.74775	0
Tomm70a	0.214561	0.397126	*Wasf3*	-2.73122	0
Vdac2	0.196049	0.376079			
Vdac3	0.186735	0.371458			
Osbpl5	0.276122	0.368455			
Fundc1	0.28507	0.366784			
Vdac1	0.194128	0.342657			
Bcl2	-0.36003	0.335311			
Stx17	-0.17081	0.32546			

Supplementary Table 2. Baseline clinical and demographic data of alcohol-associated liver disease patients in **Fig. 2f-i, 2m**

**Variables**	**Alcohol-associated liver disease (N=5)**
Age (Yrs)	40.8±7.7
Sex	3 male / 2 female
Hemoglobin (g/dl)	7.7±0.4
White blood cell counts (x10^3/mm^3)	13.8±7.05
Platelet counts (x10^3/mm^3)	83.2±48.7
Total bilirubin (mg/dl)	32.4±12.6
Aspartate aminotransferase (AST, U/L)	125.6±19.5
Alanine aminotransferase (ALT, U/L)	52.4±13.8
Alkaline phosphatase (U/L)	137.8±42.8
Albumin (g/dl)	3.34±0.49
Creatinine (mg/dl)	1.64±0.76
MELD Score	37.4±4.6

Supplementary Table 3. Primers used for qPCR analysis in the study

Gene-Species	Forward Primer	Reverse Primer
Pdk1-Mouse	CACCACGCGGACAAAGG	GCCCAGCGTGACGTGAA
Pdk2-Mouse	CCCCGTCCCCGTTGTC	TCGCAGGCATTGCTGGAT
Pdk3-Mouse	GGAGCAATCCCAGCAGTGAA	TGATCTTGTCCTGTTTAGCCTTGT
Pdk4-Mouse	CCATGAGAAGAGCCCAGAAGA	GAACTTTGACCAGCGTGTCTACAA
36B4-Mouse	ACCTCCTTCTTCCAGGCTTT	CTCCAGTCTTTATCAGCTGC
PDK1-Human	TTTACCCCCCTATTCAAGTTCATGT	CGGTCACTCATCTTCACAGTCAA
PDK2-Human	GGTGGTCAAAGATGCCTACGA	CAGGTCAGGTGAGGCCATGT
PDK3-Human	CCAGCCTGGAGCCTACCA	CGGGAAATTGGCAAACCATA
PDK4-Human	GTATGTTCCTTCTCACCTCCATCA	TGTTGCCCGCATTGCAT
GAPDH-Human	CTGGGCTACACTGAGCACC	AAGTGGTCGTTGAGGGCAATG

Supplementary Table 4. Primary antibodies used for immunoblot analysis in the study

Antibody	Source	Identifier	Dilution
COX IV	Abcam	Cat# ab16056	1:1000
FLAG	Cell signaling	Cat# 2368	1:1000
GAPDH; Clone 14C10	Cell signaling	Cat# 2118	1:1000
GRP75; Clone D-9	Santa Cruz	Cat# sc133137	1:1000
Hemagglutinin (HA); Clone F-7	Santa Cruz	Cat# sc7392	1:1000
HSP90	Cell signaling	Cat# 4874	1:1000
IP3R1; Clone E-8	Santa Cruz	Cat# sc-271197	1:1000
IP3R2; Clone A-5	Santa Cruz	Cat# sc-398434	1:1000
PDH; Clone C54G1	Cell signaling	Cat# 3205	1:1000
PDI; Clone RL90	Abcam	Cat# ab2792	1:1000
PDK1; Clone C47H1	Cell signaling	Cat# 3820	1:1000
PDK2; Clone S-15	Santa Cruz	Cat# sc-100534	1:1000
PDK3; Clone A-4	Santa Cruz	Cat# sc-365378	1:1000
PDK4 (for human)	Novus biological	Cat# NBP1-54723	1:500
PDK4 (for mouse); Clone EPR19727-245	Abcam	Cat# ab214938	1:1000
phosho-PDHE1a (Ser300)	Sigma-Aldrich	Cat# AP1064	1:10000
phosho-Ser/Thr	Cell signaling	Cat# 9631	1:1000
TOM20	Santa Cruz	Cat# sc-11415	1:1000
VDAC1	Abcam	Cat# ab15895	1:1000
β-Actin; Clone AC-15	Sigma	Cat# A5441	1:1000
β-Tubulin; Clone H3	ABM	Cat# ABM-G098	1:1000
OXPHOS complex	Abcam	Cat# ab110413	1:1000
Cyp2E1	Enzo	Cat# BML-CR3271	1:1000
SOD2; Clone D9V9C	Cell signaling	Cat# 13194	1:1000
Catalase	R&D systems	Cat# AF3398	1:1000
GPX4; Clone EPNCIR144	Abcam	Cat# ab125066	1:1000
