# Supplementary material for: Enhanced Ca2+-channeling complex formation at the ER-mitochondria interface underlies the pathogenesis of alcohol-associated liver disease
Source: Nat Commun. 2023 Mar 27;14:1703. doi: 10.1038/s41467-023-37214-4 (PMC10042999; doi:10.1038/s41467-023-37214-4)
Supplement: Supplementary file 3 — Reporting Summary [file 41467_2023_37214_MOESM3_ESM.pdf]

nature portfolio

Corresponding author(s): In-Kyu Lee
Suthat Liangpunsakul

Last updated by author(s): Jan 26, 2023

# Reporting Summary

Nature Portfolio wishes to improve the reproducibility of the work that we publish. This form provides structure for consistency and transparency in reporting. For further information on Nature Portfolio policies, see our Editorial Policies and the Editorial Policy Checklist.

## Statistics

For all statistical analyses, confirm that the following items are present in the figure legend, table legend, main text, or Methods section.

| n/a | Confirmed | |
|---|---|---|
| ☐ | ☒ | The exact sample size (*n*) for each experimental group/condition, given as a discrete number and unit of measurement |
| ☒ | ☐ | A statement on whether measurements were taken from distinct samples or whether the same sample was measured repeatedly |
| ☐ | ☒ | The statistical test(s) used AND whether they are one- or two-sided<br>*Only common tests should be described solely by name; describe more complex techniques in the Methods section.* |
| ☒ | ☐ | A description of all covariates tested |
| ☐ | ☒ | A description of any assumptions or corrections, such as tests of normality and adjustment for multiple comparisons |
| ☐ | ☒ | A full description of the statistical parameters including central tendency (e.g. means) or other basic estimates (e.g. regression coefficient) AND variation (e.g. standard deviation) or associated estimates of uncertainty (e.g. confidence intervals) |
| ☐ | ☒ | For null hypothesis testing, the test statistic (e.g. *F*, *t*, *r*) with confidence intervals, effect sizes, degrees of freedom and *P* value noted<br>*Give P values as exact values whenever suitable.* |
| ☒ | ☐ | For Bayesian analysis, information on the choice of priors and Markov chain Monte Carlo settings |
| ☒ | ☐ | For hierarchical and complex designs, identification of the appropriate level for tests and full reporting of outcomes |
| ☐ | ☒ | Estimates of effect sizes (e.g. Cohen's *d*, Pearson's *r*), indicating how they were calculated |

*Our web collection on statistics for biologists contains articles on many of the points above.*

## Software and code

Policy information about availability of computer code

| | |
|---|---|
| Data collection | Software used for data collection: Fluorescence microscopy (MetaXpress version 6.6.3.55; Molecular Devices), Histological analysis (VS200 ASW; Olympus), Immunoblot (iBright Imaging system; Thermofisher Scientific), Fluorescence intensity (SoftMax Pro version 5.4.5; Molecular Devices), Real-Time PCR (QuantStudio Real-Time PCR software v1.3; Applied Biosystems), Mitochondrial oxygen consumption rate (Wave 2.6.3; Agilent technologies). |
| Data analysis | Software used for data analysis: ImageJ 1.53q, MetaXpress analysis software version 6.5.4.532, GraphPad Prism 8, GIMP software 2.10, Sequest program version 2.4.1.15, Scaffold software version 4.11.0. |

For manuscripts utilizing custom algorithms or software that are central to the research but not yet described in published literature, software must be made available to editors and reviewers. We strongly encourage code deposition in a community repository (e.g. GitHub). See the Nature Portfolio guidelines for submitting code & software for further information.

## Data

Policy information about availability of data

All manuscripts must include a data availability statement. This statement should provide the following information, where applicable:

- Accession codes, unique identifiers, or web links for publicly available datasets
- A description of any restrictions on data availability
- For clinical datasets or third party data, please ensure that the statement adheres to our policy

All the supporting data are included in the paper and supplementary information file. Materials and their sources are provided in the Methods. Source data are provided with this paper. The mass spectrometry proteomics data have been deposited to the ProteomeXchange Consortium via the PRIDE partner repository with the dataset identifier PXD039478 and 10.6019/PXD039478.

## Human research participants

Policy information about studies involving human research participants and Sex and Gender in Research.

| | |
|---|---|
| Reporting on sex and gender | Liver tissues from 3 male/2 female patients with alcohol-associated liver disease were analyzed. Healthy control liver tissues were recieved with a de-identified data. |
| Population characteristics | Age (Yrs) 40.8±7.7 |
| Recruitment | Samples were received from the source without any biased selection. |
| Ethics oversight | The use of human liver tissues of healthy controls and patients with ALD were approved by the Indiana University Institutional Review board under the study protocol 1712511018 and 1011004278. |

Note that full information on the approval of the study protocol must also be provided in the manuscript.

# Field-specific reporting

Please select the one below that is the best fit for your research. If you are not sure, read the appropriate sections before making your selection.

☒ Life sciences ☐ Behavioural & social sciences ☐ Ecological, evolutionary & environmental sciences

For a reference copy of the document with all sections, see nature.com/documents/nr-reporting-summary-flat.pdf

# Life sciences study design

All studies must disclose on these points even when the disclosure is negative.

| | |
|---|---|
| Sample size | Based on a priori knowledge related to the study (PMID: 23449255, 32544093), sample size was calculated accordingly. A reasonable sample size was estimated so as to perform valid statistical analysis and to ensure reproducibility of the results. Exact sample size (n number) used to calculate the statistical analysis are provided for each figure in its respective figure legends. |
| Data exclusions | No data were excluded from the analyses. |
| Replication | At least 3 independent experiments perform for all the analysis (except for supplementary Figure 4A and 4B, only two independent experiments were performed). All repeated experiments gave similar results. |
| Randomization | For in vivo study, mice of the same age were randomly allocated to experimental groups before the start of the experiment. All samples collected from each individual animal in the groups were analyzed together for each experiment using the same processing and analysis method. |
| Blinding | Investigators were blinded during data acquisition and analysis for in vivo experiments. Blinding was not necessary for the in vitro experiments because same parameter and analysis method was applied to all the experimental conditions during data acquisition and analysis. |

# Reporting for specific materials, systems and methods

We require information from authors about some types of materials, experimental systems and methods used in many studies. Here, indicate whether each material, system or method listed is relevant to your study. If you are not sure if a list item applies to your research, read the appropriate section before selecting a response.

## Materials & experimental systems

| n/a | Involved in the study |
|---|---|
| ☐ | ☒ Antibodies |
| ☐ | ☒ Eukaryotic cell lines |
| ☒ | ☐ Palaeontology and archaeology |
| ☐ | ☒ Animals and other organisms |
| ☒ | ☐ Clinical data |
| ☒ | ☐ Dual use research of concern |

## Methods

| n/a | Involved in the study |
|---|---|
| ☒ | ☐ ChIP-seq |
| ☒ | ☐ Flow cytometry |
| ☒ | ☐ MRI-based neuroimaging |

# Antibodies

**Antibodies used**

Primary antibodies used in in situ PLA experiments were anti-rabbit VDAC1 (Abcam, ab15895; 1:100 dilution), anti-mouse monoclonal IP3R1 (Santa Cruz; sc-271197; Clone E8; 1:50 dilution), anti-mouse monoclonal GRP75 (Santa Cruz, sc133137; Clone D9; 1:50 dilution), anti-rabbit IP3R1 (Invitrogen, PA1-901; 1:100 dilution) and anti-rabbit FLAG (Cell signaling, #2368; 1:100 dilution).
Primary and secondary antibodies used in immunofluorescence experiments were anti-rabbit TOM20 (Santa Cruz, sc-11415; 1:100 dilution) and anti-mouse monoclonal PDI (Abcam, ab2792; Clone RL90; 1:100 dilution). Secondary antibodies conjugated with fluorophores Alexa Fluor 488 (Invitrogen, A11001; 1:100 dilution)/ Alexa Fluor 568 (Invitrogen, A11011; 1:100 dilution).
Primary antibodies used in immunoblotting experiments were COX IV (Abcam, Cat# ab16056; 1:1000 dilution), FLAG (Cell signaling, Cat# 2368; 1:1000 dilution), GAPDH (Cell signaling, Cat# 2118; Clone 14C10; 1:1000 dilution), GRP75 (Santa Cruz, sc133137; Clone D-9; 1:1000 dilution), Hemagglutinin (HA) (Santa Cruz, Cat# sc7392; Clone F-7; 1:1000 dilution), HSP90 (Cell signaling, Cat# 4874; 1:1000 dilution), IP3R1 (Santa Cruz, Cat#sc-271197; Clone E-8; 1:1000 dilution), IP3R2 (Santa Cruz, Cat# sc-398434; Clone A-5; 1:1000 dilution), PDH (Cell signaling, Cat# 3205; Clone C54G1; 1:1000 dilution), PDI (Abcam, Cat# ab2792; Clone RL90; 1:1000 dilution), PDK1 (Cell signaling, Cat# 3820; Clone C47H1; 1:1000 dilution), PDK2 (Santa Cruz, Cat# sc-100534; Clone S-15; 1:1000 dilution), PDK3 (Santa Cruz, Cat# sc-365378; Clone A-4; 1:1000 dilution), PDK4 (for detection in human samples) (Novus biological, Cat# NBP1-54723; 1:500 dilution), PDK4 (for detection in mouse samples) (Abcam, Cat# ab214938; Clone EPR19727-245; 1:1000 dilution), phosho-PDHE1a (Ser300) (Sigma-Aldrich, Cat# AP1064; 1:10000 dilution), phosho-Ser/Thr (Cell signaling, Cat# 9631; 1:1000 dilution), TOM20 (Santa Cruz, Cat# sc-11415; 1:1000 dilution), VDAC1 (Abcam, Cat# ab15895; 1:1000 dilution), β-Actin (Sigma, Cat# A5441; Clone AC-15; 1:1000 dilution), β-Tubulin (ABM, Cat# ABM-G098; Clone H3; 1:1000 dilution), OXPHOS complex (Abcam, Cat# ab110413; 1:1000 dilution), Cyp2E1 (Enzo, Cat# BML-CR3271; 1:1000 dilution), SOD2 (Cell signaling, Cat# 13194; Clone D9V9C; 1:1000 dilution), Catalase (R&D systems, Cat# AF3398; 1:1000 dilution), GPX4 (Abcam, Cat# ab125066; Clone EPNCIR144; 1:1000 dilution).

**Validation**

anti-COXIV validated for western blot (WB) and tested to detect in mouse by the manufacturer (https://www.abcam.com/cox-iv-antibody-mitochondrial-loading-control-ab16056.html); anti-FLAG validated for WB by the manufacturer (https://www.cellsignal.com/products/primary-antibodies/dykddddk-tag-antibody-binds-to-same-epitope-as-sigma-s-anti-flag-m2-antibody/2368), tested for immunofluorescence (IF) (PMID: 28076796); anti-GAPDH validated for WB and tested to detect in mouse, human by the manufacturer (https://www.cellsignal.com/products/primary-antibodies/gapdh-14c10-rabbit-mab/2118); anti-GRP75 validated for WB, immunoprecipitation (IP), IF and tested to detect in mouse, human by the manufacturer (https://www.scbt.com/p/grp-75-antibody-d-9); anti-HA is tested for detection of proteins containing the HA tag by WB, IP, IF by the manufacturer (https://www.scbt.com/p/ha-probe-antibody-f-7); anti-HSP90 validated for WB and tested to detect in mouse by the manufacturer (https://www.cellsignal.com/products/primary-antibodies/hsp90-antibody/4874); mouse anti-IP3R1 validated for WB, IF and tested to detect in mouse, human by the manufacturer (https://datasheets.scbt.com/sc-271197.pdf); rabbit anti-IP3R1 validated for WB, IF and tested to detect in mouse by the manufacturer (https://www.thermofisher.com/antibody/product/IP3-Receptor-1-Antibody-Polyclonal/PA1-901); anti-IP3R2 validated for WB and tested to detect in mouse by the manufacturer (https://www.scbt.com/p/ip3r-ii-antibody-a-5); anti-PDH validated for WB and tested to detect in mouse by the manufacturer (https://www.cellsignal.com/products/primary-antibodies/pyruvate-dehydrogenase-c54g1-rabbit-mab/3205); anti-PDI validated for WB, IF and tested to detect in mouse by the manufacturer (https://www.abcam.com/products/primary-antibodies/p4hb-antibody-rl90-ab2792.html); anti-PDK1 validated for WB and tested to detect in mouse, human by the manufacturer (https://www.cellsignal.com/products/primary-antibodies/pdhk1-c47h1-rabbit-mab/3820); ); anti-PDK2 validated for WB and tested to detect in mouse, human by the manufacturer (https://datasheets.scbt.com/sc-100534.pdf); anti-PDK3 validated for WB and tested to detect in mouse (PMID: 34552205), human by the manufacturer (https://datasheets.scbt.com/sc-365378.pdf); anti-PDK4 validated for WB and tested to detect in human by the manufacturer (https://www.novusbio.com/products/pdk4-antibody_nbp1-54723); anti-PDK4 validated for WB and tested to detect in mouse by manufacturer (https://www.abcam.com/nav/primary-antibodies/rabbit-monoclonal-antibodies/pdk4-antibody-epr19727-245-ab214938.html) and validated using PDK4 knockout animals in this paper; anti-phospho-PDHE1a (Ser300) validated for WB and tested to detect in mouse (PMID: 33826891); anti- phospho-Ser/Thr validated for WB, IP and tested to detect phospho-serine or threonine by the manufacture (https://www.cellsignal.com/products/primary-antibodies/phospho-ser-thr-phe-antibody/9631) and in the previous report (PMID: 25721419); anti-TOM20 validated for IF in mouse in the previous report (PMID: 35969774). (https://www.abcam.com/vdac1porin-antibody-mitochondrial-loading-control-ab15895.html).
anti-VDAC1 validated for WB, IF and tested to detect in mouse (this paper, using siRNA), human by the manufacturer (https://www.abcam.com/vdac1porin-antibody-mitochondrial-loading-control-ab15895.html); anti-β-Actin validated for WB and tested to detect in mouse by the manufacturer (https://www.sigmaaldrich.com/KR/ko/product/sigma/a5441); anti-β-Tubulin validated for WB and tested to detect in mouse by the manufacturer (https://www.abmgood.com/g098-1mgname.html); anti-OXPHOS complex validated for WB and tested to detect in mouse by the manufacturer (https://www.abcam.com/total-oxphos-rodent-wb-antibody-cocktail-ab110413.html); anti-Cyp2E1 validated for WB and tested to detect in mouse (this paper, by treatment of different doses of ethanol); anti-SOD2 validated for WB and tested to detect in mouse by the manufacturer (https://www.cellsignal.com/products/primary-antibodies/sod2-d9v9c-rabbit-mab/13194); anti-Catalase validated for WB and tested to detect in mouse by the manufacturer (https://www.rndsystems.com/products/human-mouse-rat-catalase-antibody_af3398?gclid=Cj0KCQiA8aOeBhCWARIsANRFrQEJUVoTZaTLy7AIZUMZvI6PsyhcxZjUmDB3TGuhYpCF78Ym4ycw5zMaAhqEEALw_wcB&gclsrc=aw.ds); anti-GPX4 validated for WB and tested to detect in mouse by the manufacturer (https://www.abcam.com/Glutathione-Peroxidase-4-antibody-EPNCIR144-ab125066.html?gclsrc=aw.ds|aw.ds&gclid=Cj0KCQiA8aOeBhCWARIsANRFrQHWe5-CkZr_VDYa91w9PKQqFLwLVYkWKFvAFjRCCkvdJW79vB1ZHREaAjOqEALw_wcB).

## Eukaryotic cell lines

Policy information about cell lines and Sex and Gender in Research

| | |
|---|---|
| Cell line source(s) | AML12 cells (CRL-2254; ATCC) were purchased from ATCC. |
| Authentication | AML12 cells were not authenticated. |
| Mycoplasma contamination | Cells were not contaminated with mycoplasma. |
| Commonly misidentified lines (See ICLAC register) | No misidentified cell lines were used in this study |

## Animals and other research organisms

Policy information about studies involving animals; ARRIVE guidelines recommended for reporting animal research, and Sex and Gender in Research

| | |
|---|---|
| Laboratory animals | 10-12 weeks old wild-type (Pdk4+/+), Pdk4 knockout (Pdk4-/-) and hepatocyte-specific Pdk4 knockout (Pdk4HepΔ), Pdk4 flox, Albumin-Cre mice (mus musculus) of C57BL/6J background were used in this study. |
| Wild animals | The study did not involve wild animals. |
| Reporting on sex | Only male mice were used in this study. |
| Field-collected samples | The study did not involve samples collected from the field. |
| Ethics oversight | All experiments were approved by the Institutional Animal Care and Use Committee of Kyungpook National University and Indiana University. |

Note that full information on the approval of the study protocol must also be provided in the manuscript.
